# The benefit of the bismuth add-on to the 2-week clarithromycin-based triple regimen for *Helicobacter pylori* eradication: a propensity score-matched retrospective study

**DOI:** 10.1186/s13099-023-00539-y

**Published:** 2023-03-19

**Authors:** Da Wit Shin, Dae Young Cheung, Ji Hee Song, Kyungseok Choi, Jihye Lim, Han Hee Lee, Jin Il Kim, Soo-Heon Park

**Affiliations:** Department of Internal Medicine, Yeouido St. Mary’s Hospital, The Catholic University of Korea College of Medicine, 63-10, Yeuongdeungpogu, Seoul, 07345 Korea

**Keywords:** Bismuth, Helicobacter pylori eradication

## Abstract

**Background:**

Bismuth salt is bacteriostatic and bactericidal against *Helicobacter pylori* (*H. pylori*). Little is known about the benefit of bismuth itself. Recently in Korea, government regulation changed to allow bismuth add-on to conventional triple eradication regimens. Study aimed the additional benefit of the bismuth add-on to the 2-week clarithromycin-based triple regimen for *H. pylori* eradication.

**Methods:**

A single-centered retrospective review of electronic medical records was conducted in Seoul, Korea. Treatment-naïve *H. pylori* infected subjects treated with the clarithromycin-based triple regimen were consecutively enrolled. After propensity score matching, 118 subjects who were treated with rabeprazole 20 mg, amoxicillin 1 g, and clarithromycin 500 mg twice daily for 14 days (PAC) and matched 118 subjects with PAC plus bismuth subcitrate potassium 300 mg twice daily for 14 days (PACB) were included in the final analysis. The primary endpoint was the eradication success rates in each group.Article title: Kindly check and confirm the edit made in the article title.Yes, I agree with the article title.

**Results:**

The eradication success rates were 91.5% (86.4–96.6%) for PACB regimen and 81.4% (74.2–88.5%) for PAC in the intention-to-treat analysis, and 97.3% (94.2–100%) for PACB and 88.1% (81.9–94.3%) for PAC in the per-protocol analysis. The relative risk of eradication failure for PACB over PAC was calculated as 0.184 (0.0492–0688, p value = 0.005) in multiple regression logistic analysis. Compliance and adverse event incidence were not different between the two groups.Author names: Please confirm if the author names are presented accurately and in the correct sequence (given name, middle name/initial, family name). Author 1 Given name: [Da Wit], Last name: [Shin]. Author 2 Given name: [Dae Young], Last name: [Cheung]. Author 3 Given name: [Ji Hee], Last name: [Song]. Author 4 Given name: [Fan Hee], Last name: [Lee]. Author 5 Given name: [Jin Il], Last name: [Kim]. Yes. I found the names presented are accurate and in the correct sequence. Author 1 Given name: [Da Wit], Last name: [Shin].Author 2 Given name: [Dae Young], Last name: [Cheung].Author 3 Given name: [Ji Hee], Last name: [Song].Author 6 Given name: [Han Hee], Last name: [Lee].Author 7 Given name: [Jin Il], Last name: [Kim].

**Conclusion:**

The bismuth add-on to the 2-week clarithromycin-based triple regimen increased the eradication success rate.

## Introduction

Since the discovery of *Helicobacter pylori (H. pylori)*, various antibiotics have been studied for eradication, and so far, amoxicillin and clarithromycin are recognized as having the most excellent antimicrobial effects against *H. pylori* [1–3]. For this reason, a triple combination regimen consisting of proton pump inhibitor (PPI), amoxicillin, and clarithromycin has been used as a standard treatment. However, as the frequency of resistant strains to clarithromycin increases, the eradication rate of the triple regimen gradually decreases. In Korea, clarithromycin resistance was not as high as 9% in 1995 [4], but it increased to 17.8% according to the latest study conducted by the Korean Society of Helicobacter and Upper Gastrointestinal Disease Research Group in 2018 [5]. For this reason, to overcome the decrease in the antibacterial efficacy due to the increase in clarithromycin resistance, guidelines recommends the extension of dosing period is from 7 to 14 days, or a quadruple regimen containing clarithromycin and metronidazole simultaneously, or bismuth-containing quadruple regimen as a first-line treatment [6, 7].

It is well known that bismuth has a bacteriostatic or bactericidal effect against *H. pylori* [8, 9]. Bismuth-containing quadruple regimen has traditionally been recommended as a second-line therapy after failure of the first-line eradication therapy, but in 2017 European consensus guidelines, it was suggested to be used first-line in regions with high clarithromycin resistance [10]. However, the bismuth-containing quadruple regimen is neither suitable nor feasible for the first-line treatment for all infected patients. The four-times drug taking for tetracycline is quite complicated and when used in combination with metronidazole, tetracycline often results in significant inconveniences such as a metallic taste. In a study of modified bismuth quadruple regimen that used amoxicillin instead of tetracycline, the eradication success rate as well as the incidence of adverse events was equivalent to the conventional bismuth quadruple regimen [11]. That suggested that the additional benefit of bismuth was effective not only with tetracycline but also in combination with amoxicillin. The advantage of bismuth is that it works synergistically with antibiotics, possibly can overcome the resistance of clarithromycin or quinolone [10], and does not develop resistance to *Helicobacter pylori* regardless of the duration of use or repetition.

Regarding the benefit of bismuth, most studies have been conducted comparing bismuth-containing quadruple regimen and other combination regimens [12–14]. Those study designs are hard to tell the impact of bismuth separately. In this study, the authors investigated the clinical outcomes of a 2-week clarithromycin-based triple regimen with a bismuth add-on compared to that regimen without a bismuth add-on.

### Patients and methods

#### Patients

**Fig. 1:**
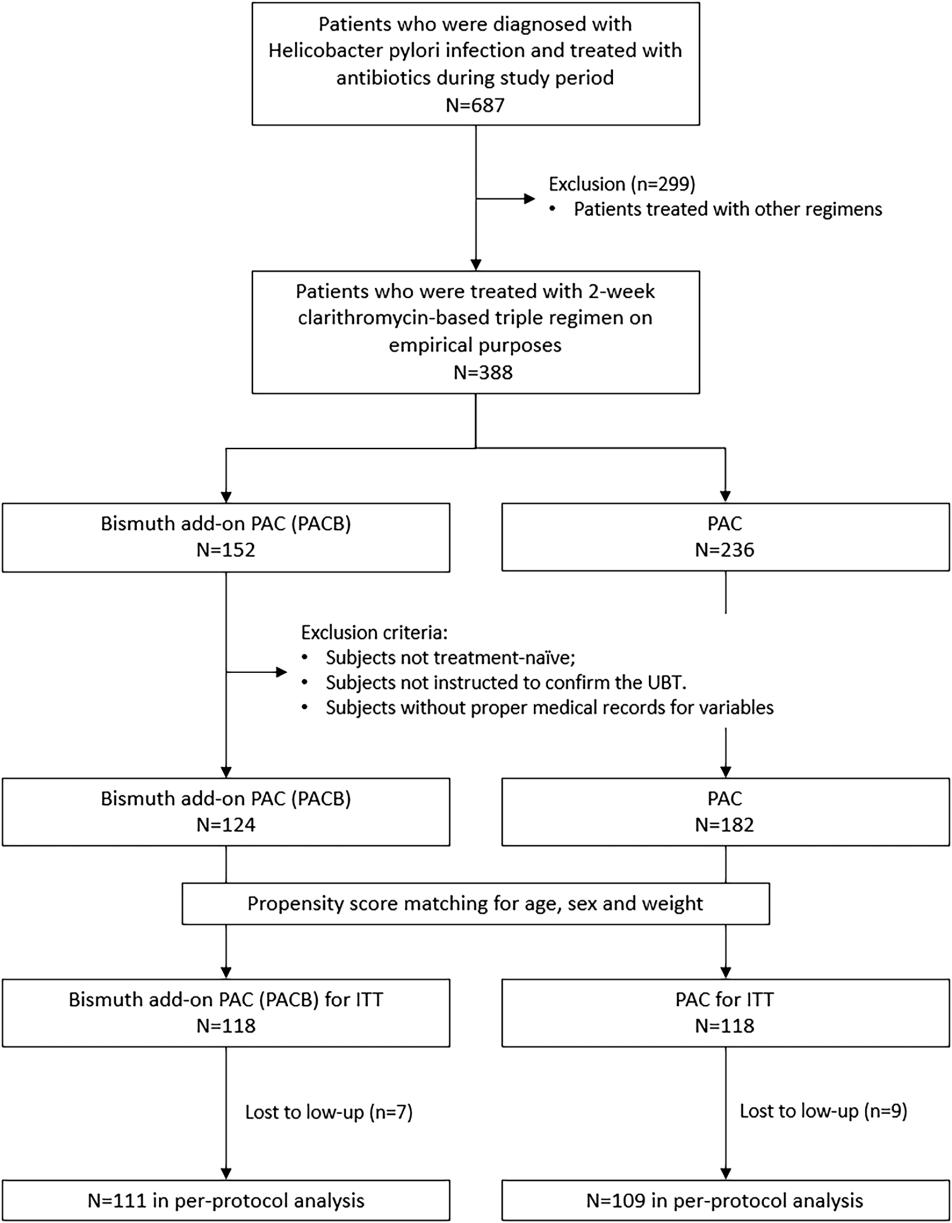
Flow chart of subjects’ enrollment. PAC indicates 2-week combination regimen consists of proton pump inhibitor, amoxicillin, and clarithromycin. PACB indicated PAC regimen with bismuth add-on. ITT, intention-to-treat

This study is a single-centered retrospective review of electronic medical records. From October 2018 to October 2022, a total of 687 patients were treated for *H. pylori* infection at the Catholic University of Korea Yeouido St. Mary's Hospital. All patients were diagnosed with *H. pylori* infection by histological examination with routine hematoxylin-eosin staining and Warthin-Starry stating of the biopsied gastric mucosa. Among those, 236 subjects were administered with a conventional 2-week clarithromycin-based triple regimen (PAC), and 152 subjects were with PAC plus bismuth add-on (PACB). PAC regimen consisted of rabeprazole 20 mg twice daily, amoxicillin 1.0 g twice daily and clarithromycin 500 mg twice daily for 14 days. PACB regimen consisted of PAC and bismuth subcitrate potassium 300 mg (Denol tablets, Green Cross Corp, Korea) twice daily. For the final analysis, subjects who met the followings were enrolled; *H. pylori-*infected patients with no history of prior treatment; the eradication regimen was chosen on the purpose of empirical treatment [6]; the confirmation of eradication result was done by ^13^C-urea breath test (UBT); the medical records for baseline characteristics and adverse events presented. Records from subjects with a history of esophageal or gastric surgery or an allergy to any eradication drug, or any severe comorbidities were excluded. Finally, 124 subjects with PACB regimen and 182 subjects with PAC regimen were pooled for propensity score matching (PSM). Given that confounders such as age and sex may significantly influence the drug metabolism and eradication efficacy, subjects in the PACB group were a 1:1 propensity score matched (PSM) to subjects in the PAC group. One hundred and eighteen subjects for each group were matched for final comparison. (Fig. 1) This study was conducted with approval from the Clinical Research Ethics Committee of the Catholic University of Korea Yeouido St. Mary's Hospital. (IRB approval number, SC22RISI0039) The board waived the requirement for informed consent for retrospective review and analysis.

Article structure: Kindly check whether the section headings have been identified correctly and amend if any.Yes, I agree with the section headings. 

### Intervention

Subjects were treated with clarithromycin-based tiple regimen with bismuth (PACB group, n = 118) or without bismuth (PAC group, n = 118) for 14 days. All subjects were given two probiotics supplements during the 2-week treatment period to reduce the possible adverse events. Probiotics consisted of *Lactobacillus casei* variety *rhamnosus* 250 mg (Ramnos granule, Hanwha pharmaceutical, Korea) twice daily and *Bacillus Subtilis·Enterococcus faecium* culture 250 mg (Medilac-DS Enteric coated capsule, Hanmi pharmaceutical, Korea) twice daily. Probiotics supplements were indicated to be taken at the time of eradication regimen dosing, regardless of mealtime.

### Study outcomes

The primary endpoint of this study was the eradication success rates of PACB and PAC regimens. Eradication success was defined as a negative ^13^C-urea breath test (UBT, Heliview; MediChems, Seoul, Korea). The cutoff value was 2.0‰. UBT was performed at least 4 weeks away from any antibiotics or proton pump inhibitors usage including eradication regimens. The second endpoints were the compliance and the incidence of adverse events (AEs). The drug compliance and occurrence of any adverse events were investigated based on the contents of medical records. Questions about the drug compliance and occurrence adverse events were asked by the attending physician during the outpatient interviews at the time of completion of treatment or UBT result confirmation. Proper compliance was judged when the drugs were taken > 85% doses (24/28 doses, 12/14 days). The severity of adverse events was arbitrarily divided into three levels: none, present but tolerable, and intolerable requiring stop dosing.

### Statistics

Three features, age, sex, and body weight were utilized to estimate the propensity score using a logistic regression model. Matching was then performed based on the estimated propensity score employing the nearest neighbor approach with a caliper width of 0.20. Propensity score estimation and matching were done by the SPSS package (KoreaPlus Statistics Embedded on SPSS statistics 26 standard, Datasolution Inc, Seoul, Korea).

Statistical analysis was performed using the SPSS statistical analysis package. Nominal variables are presented as absolute frequencies with 95% confidential intervals, and continuous variables are as the means ± standard deviations (SDs). The results were analyzed using the Chi-square test and Fisher’s exact test for nominal variables and Student's t-test for continuous variables. When p < 0.05, it was judged to be statistically significant.

Intention-to-treat (ITT) analysis included all propensity score matched subjects. Subjects who did not complete the urea breath test after eradication therapy or lost to follow-up were considered treatment failure in the ITT analysis. The per-protocol (PP) analysis only included the subjects who completed the treatment and UBT test.

## Results

### Baseline characteristics of enrolled subjects

A total of 236 subjects were finally enrolled for final analysis. The flow for subject enrollment is depicted in Fig. 1. The demographic information and clinical characteristics of enrolled subjects in PACB and PAC groups are summarized in Table 1. The mean ages of the PACB group and the PAC group were 60.1 (± 12.7) years and 59.4 (± 12.4) years, respectively. The proportion of males was 61.9% and 58.5% for the PACB and PAC groups, respectively. The mean body weight was 63.9 (± 10.2) Kg and 63.8 (± 9.5) Kg for the PACB group and the PAC group, respectively. Age, sex and weight were not different between two groups. Regarding the smoking and presence of comorbidities, two groups showed comparable distribution. Alcohol users were more prevalent in PACB group than PAC group, 44.9% and 29.1%, respectively (p-value = 0.024). The endoscopic diagnoses for Helicobacter pylori infection were not different between the groups Table 2.

**Table 1:** Baseline Characteristics of patients according to designated groups

	PACB (n = 118)	PAC (n = 118)	p-value
Age (mean, SD)	60.1 (12.7)	59.4 (12.4)	0.693
Sex			0.595
Male (n,%)	73 (61.9)	69(58.5)	
Female (n,%)	45(38.1)	49 (41.5)	
Body Weight (mean, SD)	63.9 (10.2)	63.8 (9.5)	0.939
Smoking			0.496
Current	24 (20.3)	21 (17.9)	
Never	84 (71.2)	90 (76.9)	
Ex	10 (8.5)	6 (5.1)	
Alcohol			0.024
Current	53 (44.9)	34 (29.1)	
Never	59 (50.0)	79 (67.5)	
Ex	6 (5.1)	4 (3.4)	
Comorbidity			
Having one or more comorbidity	49 (41.5%)	51 (43.2%)	0.792
CKD	1 (0.8%)	0	1.000
DM	14 (11.9%)	22 (18.6%)	0.148
Hypertension	18 (15.3%)	18 (15.3%)	1.000
Dyslipidemia	21 (17.8%)	21 (17.8%)	1.000
Use of aspirin or antiplatelets	1 (0.8%)	5 (4.2%)	0.213
Malignancy of other organs	2 (1.7%)	2 (1.7%)	1.000
Reason for *H.pylori* eradication			0.300
*H.pylori* gastritis	39 (33.1)	41 (34.7)	
AG-IM	16 (13.6)	23 (19.5)	
PUD	39 (33.1)	27 (22.9)	
Dysplasia or carcinoma	24 (20.3)	27 (22.9)	

PACB, clarithromycin-based 2-week triple regimen with bismuth add-on; PAC, clarithromycin-based 2-week triple regimen; CRF, chronic kidney disease; DM, diabetes mellitus; AG, atrophic gastritis; IM, intestinal metaplasia

**Table 2:** The eradication success rates of regimens

Analysis	PACB	PAC	p-value
ITT	(n = 118) 91.5%	(n = 118) 81.4%	0.023
95% CI	0.8643–0.9662	0.7423–0.8849	
PP	(n = 111) 97.3%	(n = 109) 88.1%	0.008
95% CI	0.9423–1.00 36	0.8189–0.9426	

PACB, clarithromycin-based 2-week triple regimen with bismuth add-on; PAC, clarithromycin-based 2-week triple regimen; ITT, intent-to-treat; PP, per protocol; CI, confidential interval

### The benefit of bismuth add-on to the clarithromycin-based triple regimen

The eradication success rates were 81.4% (95% CI 0.7423–0.8849) for PAC group and 91.5% (95% CI 0.8643–0.9662) for PACB group in ITT analysis (p-value = 0.023). The odds risk for treatment failure for PAC group was 2.475(95% CI 1.116–5.489). The increment of the eradication success rate of PACB group over PAC was 12.5%. Similarly in PP analysis, the eradication success rates were 88.7% (95% CI 0.8189–0.9426) for PAC group and 97.3% (95% CI 0.9423–1.0000) for PACB group (p-value = 0.008) and the odds risk for treatment failure for PAC group was 4.875 (95% CI 1.348–17.624). The increment of the eradication success rate of PACB group over PAC was 10.5%.

### Drug compliance and adverse events

**Fig. 2:**
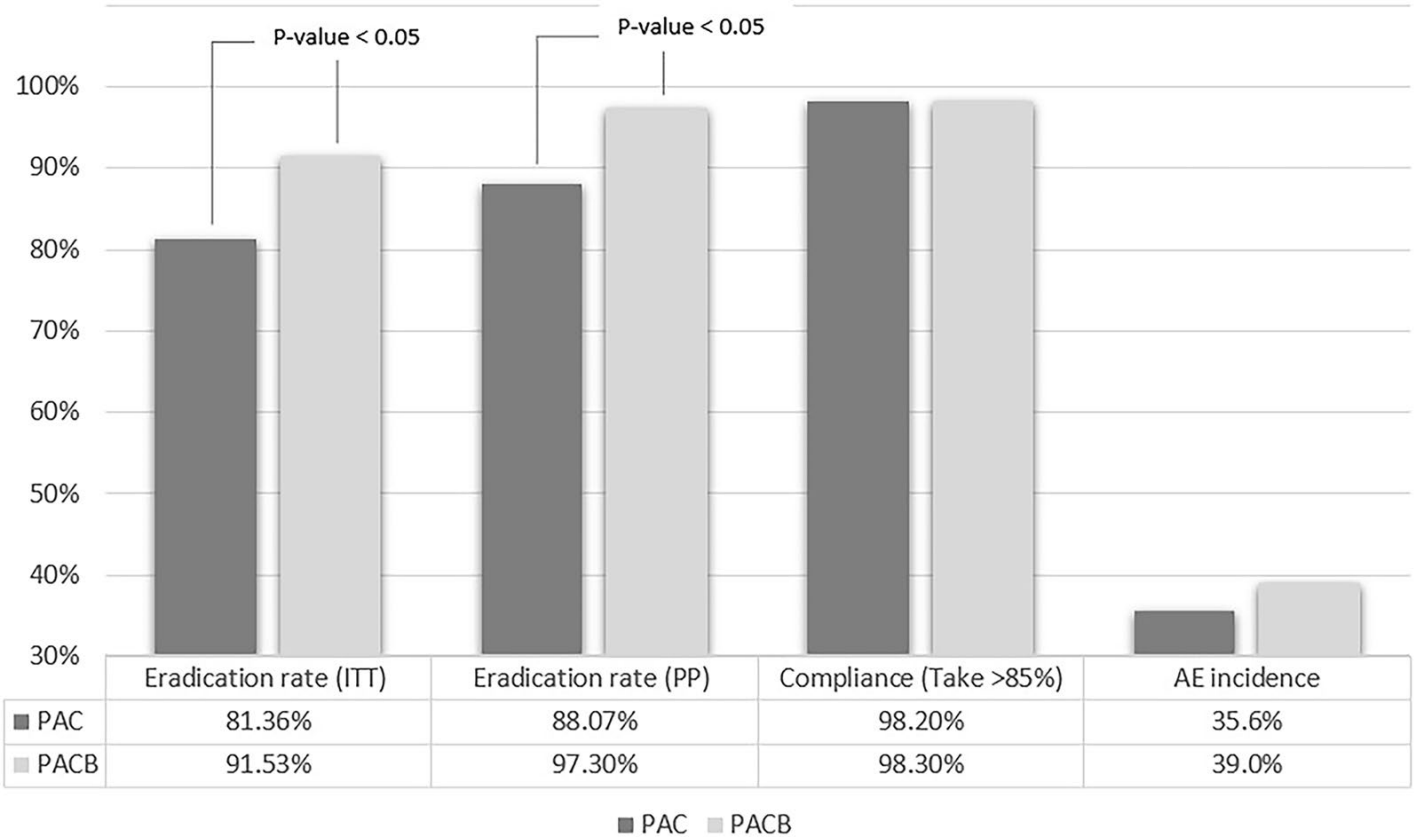
The outcomes of 2-week clarithromycin-based triple regimen with and without bismuth add-on for an empirical purpose. PAC indicates 2-week combination regimen consists of proton pump inhibitor, amoxicillin, and clarithromycin. PACB indicated PAC regimen with bismuth add-on. ITT, intention-to-treat; PP, per protocol, AE, adverse events

The adverse events occurred in one-third of the subjects and the overall adverse events rates were not different between PACB and PAC groups. The frequency and manifestation of the adverse events were described in Table 3. Most subjects described the severity of the adverse events as minimal without interfering of daily activity. Only one subject dropped taking medication due to nausea, vomiting and headache. The frequency and severity of the adverse events were not different between PAC and PACB groups. The most common adverse events were loose stool in PACB group and vague discomfort hard to describe specifically in PAC group. Loose stool and metallic taste happened more frequently in PACB group than in PAC group (p-value < 0.05). Other kinds of adverse events were not differently occurred in both groups. All adverse events subsided spontaneously and without any intervention following cessation of medication. Over 98% of subjects in both groups reported proper drug compliance. Four subjects from PACB group and 9 subjects from PAC were lost to follow-up for asking drug compliance adverse events. Study results are summarized in Fig. 2.

**Table 3:** Drug compliance and drug associated adverse events

	PACB	PAC	p-value
Incidence of AE (ITT) (/n = 118)	39.0%	35.6%	
Drug compliance	(n = 115)	(n = 109)	
Taking > 85%	113 (98.3)	107 (98.2)	0.957
Taking less than 85%	2 (1.7)	2 (1.8)	
Severity of AE	(n = 115)	(n = 109)	
None	72 (62.6)	76 (69.7)	0.347
Present but tolerable	43 (37.4)	32 (29.4)	
Intolerable and need to stop taking	0	1 (0.9)	
AE characteristics	(n = 115)	(n = 109)	
Nausea-vomiting	8 (7.1)	2 (1.8)	0.102
Loose stool	18 (15.9)	1 (0.9)	> 0.001
Constipation	1 (0.9)	0	1.000
Headache	4 (3.5)	0	0.122
Metallic taste	8 (7.1)	1 (0.9)	0.036
Indigestion	3 (2.7)	0	0.247
General weakness	1 (0.9)	0	1.000
Epigastric soreness	3 (2.7)	0	0.247
Indescribable non-specific discomfort	10 (8.8)	28 (25.7)	1.000

AE, adverse event; ITT, intention-to-treat

## Discussion

The study showed the benefit of the bismuth add-on to 2-week clarithromycin-based triple therapy with propensity matching score analysis and investigated whether the bismuth add-on can overcome the clarithromycin resistance with arithmetic calculations indirectly. The study also showed two probiotics supplements (*Lactobacillus casei* variety *rhamnosus* and *Bacillus Subtilis·Enterococcus faecium* culture) might reduce the adverse event and enhance the eradication success.

Though the socioeconomic improvement in the twentieth century markedly decreased the prevalence of *H. pylori* infection in populations in developed countries, the need for treatment for *H. pylori* infection is still valid. To eradicate *H. pylori* in human, a variety of antibiotics and acid suppressants with enhanced potency have been tried at higher doses and for prolonged durations. However, we still have no definitive regimen of acceptable and liable eradication results. On top of that, along with the increased use of antibiotics globally, we encountered antibiotics-resistant *H. pylori*, especially to clarithromycin [15]. We are in urgent need to find a new drug that can increase the eradication effect without incurring resistance.

Bismuth is not new, but well known to be safe and used for long. Though it is not a kind of antibiotics, it has a bactericidal or bacteriostatic effect on *H. pylori.* Bismuth is commonly used as a component drug with other antibiotic combinations for *H. pylori* eradication [6, 10, 16, 17]. The bismuth-containing quadruple regimen were reported to result in more than 90% eradication success rate and to be effective even in cases of clarithromycin or metronidazole resistance. This effect was maintained even when tetracycline was substituted for amoxicillin [11].

In our study, authors sought to find the benefit of bismuth add-on by comparing clarithromycin-based triple regimens with and without bismuth. Bismuth add-on increased the eradication rate from 81.4% to 91.5% in ITT analysis (p-value = 0.023) and from 88.7% to 97.3% in PP analysis (p-value = 0.008). With bismuth add-on, the odd ratio for treatment failure decreased to 1 of 2.475 in ITT analysis and 1 of 4.875 in PP analysis. Our results look compatible and supportive with previous reports about the bismuth add-on. A recent European registry study reported an eradication success rate of 88% in intention-to-treat and 94% in per-protocol analysis when bismuth was added to a 14-day standard triple-drug regimen [18]. A retrospective single-arm study by Kim et al. reported that when it was added to the 2-week standard triple regimen the final eradication success rate reached 77.1% in the subjects with clarithromycin resistance [19]. This was also a better outcome than that of conventional clarithromycin-based triple regimen for clarithromycin-resistant *H.pylori.* Horie et al. reported eradication rates of 88% and 45% with clarithromycin-based triple regime for clarithromycin-sensitive and clarithromycin-resistant cases, respectively [20].

To the question of whether the bismuth add-on can overcome the clarithromycin resistance, the answer was not directly explored in this study. However, the possibility can be estimated by arithmetic calculations using relevant data [20, 21] and the authors’ study outcomes. If we cite the recent Korean research result [21] on the clarithromycin resistance, 17.8%, and eradication outcomes from Horie’s study [20], the calculated estimation of eradication success with conventional triple therapy (PAC) in Korean population is 80.3%. This simple calculation result, 80.3%, is compatible with our result of 81.4% (95% CI 0.7423–0.8849) of eradication success rate in ITT analysis for PAC group and supportive of the reliability of our study. The effect of the bismuth add-on on the clarithromycin-resistant *H. pylori* can also be estimated by following arithmetic calculation; [91.5% of final eradication rate in ITT analysis of PACB group = prevalence of clarithromycin-resistant H. pylori infection (17.8%) x unknown eradication rate (x) + prevalence of clarithromycin-sensitive H. pylori infection in Korea (82.2%) x unknown eradication rate (y)]. With this arithmetic calculation, the “y” value is calculated in the range of 88%(20)–100%, and “y” should not be less than “x”, then consequently the range of the “x” value should be between 52.4% and 91.5%. If the proportion of clarithromycin-resistant *H. pylori* increases, the lowest estimate of the interval will also increase. This is significantly higher than the 45% result of Horie et al. and is consistent with the results of other previous studies [18, 19, 22]. We can conclude that the bismuth add-on can overcome the clarithromycin resistance. Nevertheless, the authors do not advocate the use of a bismuth-added clarithromycin-based triple regimen for clarithromycin-resistant *H.pylori* based on the results of this study. Because it is reasonable to use metronidazole if clarithromycin resistance is known. The authors would like to emphasize that the bismuth add-on can improve the overall eradication outcomes of a 2-week clarithromycin-based triple regimen in situations where an empirical treatment is required. Contrary to the results of this study, Wu et al. reported that the eradication rate could not be improved even with a bismuth add-on for the clarithromycin-based triple regimen [23]. However, Wu et al.'s study differ from the authors' study in that it was the result of administering a clarithromycin-based triple regimen for one week in an empirical condition.

One of the concerns, when we use bismuth, is compliance and possible adverse events. In the authors’ study, the proportion of subjects who responded to take over 85% of the total doses was over 98% in both PACB and PAC groups. It looks higher than that of other reports even including prospective studies in which compliance was reported around 81–98% with the standard triple regimen [24, 25]. We admit the concern about recall bias due to the retrospective design of this study. However, the authors would like to suggest another possible explanation for better compliance than expected. All subjects in this study were prescribed two probiotics supplements along with antibiotics. Probiotics including *Lactobacillus rhamnosus* GG are known to help to reduce the side effects of eradication therapies and improve compliance with therapy [26]. A meta-analysis by Dang et al. reported that higher eradication rate in probiotics supplementation groups than in control (ITT analysis: RR 1.122, 95% CI 1.086–1.159, PP analysis: RR 1.114, 95% CI 1.070–1.159) and lower incidence of adverse event (RR 0.735, 95% CI 0.598–0.902) [27]. Argues are still on the table about the beneficial strains of probiotics and the definite mechanism for these effects, a variety of probiotic strains have been listed to be helpful in *H. pylori* eradication [28].

Regarding the adverse events, over 35% of subjects reported having discomfort or adverse events during administration. The authors acknowledge the concerns and potential for recall bias and missing records when collecting adverse event data. However, again, the authors infer that the coadministration of probiotics supplements might play a role in reducing the occurrence of possible adverse events. More importantly, the limitations of the retrospective investigation were the same for both PAC and PACB groups, and there was no difference in the incidence of adverse events according to the bismuth add-on or not. Almost all subjects in PACB group experienced dark colored stool, but the change of stool color predicted though the explanation before taking the regimen was not judged to be uncomfortable. Our results were comparable to other previous prospective studies using bismuth [25, 29] and a study from China reported 28% of adverse event incidence with 2-week PACB regimen [1]. Actually tetracycline was suggested as a major reason for adverse events in the bismuth-containing quadruple regimen[24] and bismuth itself was reported to reduce the discomfort during eradication therapy [30, 31].

Regarding rescue therapy after 1st line standard triple regimen failure, recent guidelines recommend bismuth-containing quadruple regimen as the 2nd line therapy. The authors' institution uses bismuth-containing quadruple regimen as recommended or metronidazole-based triple regimen as a 2nd line if the 1st line was clarithromycin-based triple regimen. This is most likely due to clarithromycin resistance when clarithromycin-based triple regimen therapy fails, and because metronidazole-based triple regime is generally judged to be relatively convenient to take for bismuth-containing quadruple regimen. A total of 16 of the enrolled subjects were diagnosed with treatment failure after UBT examination. The results of secondary treatment for them will be further analyzed in the future.

This study has some limitations. First, due to the retrospective study design, the concern about recall bias and missed records was inevitable. However, an important issue in attempting randomized studies to measure the effects of bismuth add-on is the ethical question of the probably expected negative effects in a control group. Recently in Korea, there have been significant changes in the usage direction of bismuth. From January 2019, under ‘Ministry of Health and Welfare Drug Notification No. 2018–174 on the standards of medical care benefits and Detailed information on the application methods’, bismuth drug can be used without restrictions to eradicate *H. pylori* beyond the previously approved indications. Thanks to this situational condition, the authors could carry out a parallel comparative study with and without bismuth. In this study, the two comparison groups had nearly identical baseline characteristics regarding residence area and time of intervention. The authors adopted propensity score matching analysis to minimize possible bias in outcomes by undetected factors. Second, this is a single-centered investigation. We need a multi-centered investigation to generalize our results. Third, antibiotics susceptibility test was not employed in this study. The study design was determined by considering that an empirical therapy is performed in most clinical practices, but the authors acknowledge that the interpretation of the study results would be more precise if the information on antibiotic susceptibility were available.References: As per pubmed findings, citation details [DOI] for Reference [8, 24] have been inserted. Kindly check and confirm the inserted details.Yes, I agree with the amendment. 

## Conclusions

The bismuth add-on to 2-week clarithromycin-based triple regimen for an empirical purpose significantly increased the eradication success rate. Bismuth add-on neither worsens the compliance nor the incidence of adverse events. Probiotics supplements during the administration of the eradication regimen possibly were helpful in reducing adverse events and improving compliance and eradication outcomes.

## Data Availability

Data are available upon request from the authors.
